# Identification of genomic regions associated with cereal cyst nematode (*Heterodera avenae* Woll.) resistance in spring and winter wheat

**DOI:** 10.1038/s41598-023-32737-8

**Published:** 2023-04-11

**Authors:** Deepti Chaturvedi, Saksham Pundir, Vikas Kumar Singh, Deepak Kumar, Rajiv Sharma, Marion S. Röder, Shiveta Sharma, Shailendra Sharma

**Affiliations:** 1 Department of Genetics and Plant Breeding, Chaudhary Charan Singh University (CCSU), Meerut, Uttar Pradesh 250004 India; 2 Department of Botany, Chaudhary Charan Singh University (CCSU), Meerut, Uttar Pradesh 250004 India; 3 Scotland’s Rural College (SRUC), Peter Wilson Building, West Mains Road, Edinburgh, EH9 3JG UK; 4 Leibniz Institute of Plant Genetics and Crop Plant Research (IPK), Corrensstrasse 3, OT Gatersleben, 06466 Seeland, Germany

**Keywords:** Genetics, Plant sciences

## Abstract

Cereal cyst nematode (CCN) is a major threat to cereal crop production globally including wheat (*Triticum aestivum* L.). In the present study, single-locus and multi-locus models of Genome-Wide Association Study (GWAS) were used to find marker trait associations (MTAs) against CCN (*Heterodera avenae*) in wheat. In total, 180 wheat accessions (100 spring and 80 winter types) were screened against *H. avenae* in two independent years (2018/2019 “Environment 1” and 2019/2020 “Environment 2”) under controlled conditions. A set of 12,908 SNP markers were used to perform the GWAS. Altogether, 11 significant MTAs, with threshold value of −log10 (*p*-values) ≥ 3.0, were detected using 180 wheat accessions under combined environment (CE). A novel MTA (*wsnp_Ex_c53387_56641291*) was detected under all environments (E1, E2 and CE) and considered to be stable MTA. Among the identified 11 MTAs, eight were novel and three were co-localized with previously known genes/QTLs/MTAs. In total, 13 putative candidate genes showing differential expression in roots, and known to be involved in plant defense mechanisms were reported. These MTAs could help us to identify resistance alleles from new sources, which could be used to identify wheat varieties with enhanced CCN resistance.

## Introduction

Wheat (*Triticum aestivum* L.) is one of the most widely grown crop, cultivated on ~ 220 million ha, with a worldwide annual production of over 700 million tons, and global annual export value of around US$50 billion^1^. China was the top wheat producing country in 2022 with production volume of over 137 million metric tons followed by India, Russia, Australia, Canada, Pakistan, and Ukraine^2^. Plant-parasitic nematodes (PPNs) are a serious global problem to wheat production^3–5^. Root-knot nematodes (RKNs; *Meloidogyne* spp.), cereal cyst nematodes (CCNs; *Heterodera* spp.), and root lesion nematodes (RLNs; *Pratylenchus* spp.) are the most common PPNs responsible for reduction in crop yield worldwide^6^. *Heterodera avenae* is an economically significant PPN in temperate wheat-producing regions like North and South Africa, East and West Asia, Australia, Europe, the Indian Subcontinent, the Middle East, and North America^7^. Cultivation of susceptible cultivars is the main cause of severe yield losses due to *H. avenae* infection in majority of wheat growing regions^5,8^.

In order to reduce the threat posed by rising *H. avenae* population densities and to keep the losses incurred below economic threshold, it is crucial to develop efficient disease management strategies. Crop rotation, chemical, and biological management strategies are some of the common approaches that could help to reduce the damage caused by these nematodes to some extent. Few nematicides such as aldicarb, oxamyl, abamectin are known to be effective in decreasing the nematode population and increasing yield in wheat and barley^8–10^. Despite their benefits several nematicides are banned due to their high risk for human health and environment. Therefore, finding the host plant resistance is one of the most effective, economically and environmentally sustainable method to prevent or reduce nematode multiplication^11^.

Resistance genes against CCNs were identified in *T. aestivum* including *Cre1*^12–14^ and *Cre8*^15^. Several known genes were introgressed into cultivated wheat from wild relatives such as *Cre2*, *Cre5* and *Cre6* were transferred from *Aegilops ventricosa*^16–18^, *Cre3* and *Cre4* were introgressed from *Ae. tauschii*^19^, *Cre7* from *Ae. triuncialis*^20^ and *CreR* from rye^21,22^. *CreX* and *CreY* were pyramided into a single bread wheat line from *Ae. variabilis*^23–25^. Development of wheat cultivars resistant against nematodes is a challenging task for breeders. Raj MR1, CCNRV2, and CCNRV4 are some of the varieties in India reported to show resistance against *H. avenae*^26^. Also, some of the CIMMYT synthetic wheat derivatives e.g. CROC_1/AE, SQUARROSA (224)//OPATA showed resistance to numerous soil-borne pathogens including CCNs as well as root lesion nematode *Pratylenchus thornei*^27,28^. Wheat cultivars such as Meering, Festiguay, Molineux, Frame, Chara, and Annuello in Australia are found to be moderately resistant to *H. avenae*^29^.

Quantitative trait loci (QTLs) mapped in bi-parental crosses is restricted in allelic diversity and has limited genomic resolution whereas genome-wide association approaches offers higher resolution and also eliminate the need to develop mapping population^30^. Only few QTL studies have been conducted in wheat for *H. avenae* resistance^14,31–34^. Rapid advancement in the field of genotyping technology offered novel, cheaper and faster methods of sequencing plant genome. The presence of high-density single-nucleotide polymorphism (SNP) permits scanning of whole-genome to obtain usually small haplotype blocks that are significantly associated with quantitative trait variation and it has been widely used to detect MTAs for several yield-related traits^35,36^. So far, few GWA studies, have been conducted in wheat for *H. avenae* resistance. Mulki et al.^28^ carried out a GWAS on 332 synthetic hexaploid wheat lines genotyped with 660 Diversity Arrays Technology (DArT) and identified 17 markers loci significantly associated with CCNs and 12 with *P. neglectus.* Among these identified loci, five novel QTLs were identified for resistance to CCN on chromosomes 1D, 4D, 5B, 5D and 7D and three for *P. neglectus* on chromosomes 4A, 5B and 7B. In another study, 126 advanced CIMMYT spring wheat lines were screened for resistance against *H. aveane*, *P. neglectus* and *P. thornei*. Eleven MTAs were identified for resistance to *H. avenae*, 25 were linked with resistance against *P. neglectus* and nine MTAs for *P. thornei*. Three novel QTLs were mapped for resistance to *H. avenae* on chromosomes 5A, 6A, and 7A^37^.

The main objective of the present study was to identify novel sources of resistance to *H. avenae* in a set of hexaploid wheat of both winter and spring growth habits. Single and multi-locus GWAS models were applied in the present study to control false positives. In addition, candidate genes underlying significant MTAs were also reported. These MTAs shall provide a useful set of markers to perform Marker-Assisted Selection (MAS) based breeding in wheat.

## Materials and methods

### Association panel

The association panel used in the present study consisted of hexaploid wheat of both winter and spring growth habits. The spring wheat panel had 100 accessions and the winter wheat panel included 80 accessions. The details of all the 180 accessions with their growth habits and origin are presented in supplementary data (Supplementary Table S1A, B). The spring panel was obtained from the Genebank of IPK in Gatersleben (http://www.ipk-gatersleben.de/en/genebank/) described in Muqaddasi et al.^38^. The winter wheat panel was from a larger core collection of the Institute of Field and Vegetable Crops (Novi Sad, Serbia) described in Neumann et al.^39^.

### Genotypic data

An original set of 207 wheat accession was analyzed by using a 15K Infinium SNP array Muqaddasi et al.^38^. The development of a 15K SNP-chip and the genotyping has been carried out by TraitGenetics GmbH (http://www.traitgenetics.com). After removing the calls of failed SNPs and the SNPs with ambiguous calls, a total of 13,006 SNPs were obtained from a 15 K Infinium SNP array that is an optimized and reduced version of the 90K iSELECT SNP-chip described by Wang et al.^40^. The remaining markers were again filtered for minor allele frequency (MAF) ≥ 5% which resulted in 12,908 SNPs. This set of 12,908 markers (including both mapped as well as unmapped) was subsequently used for the analysis.

### Screening experiment

Screening experiments were carried out under controlled environmental conditions (22 ± 2 °C, 16 h light, and 8 h darkness and ~ 65% relative humidity) in growth room in the Department of Genetics and Plant Breeding, Ch. Charan Singh University, Meerut. All experiments were carried out as completely randomized design (CRD) in four different batches with a maximum of five replications over two consecutive years (Environment 1 or E1 and Environment 2 or E2). The accessions were phenotypically screened for resistance against *H. avenae* by counting the number of cyst formed on individual plant.

For inoculum preparation, soil samples (infested with *H. avenae*) were collected from an experimental field located at Hisar (latitude, 29.3815 °N, longitude, 75.5750 °E), India. Cysts were extracted by using sieves of different sizes i.e., 850 µm, 250 µm and 150 µm sizes following Cobb’s decanting and sieving method^41^. Soil samples were suspended in a beaker filled with water and stirred well and then passed through 850 µm, 250 µm and 150 µm sieves under running water. Cysts retained on 250 µm sieve were then hand-picked under a stereo-microscope and stored at 4 °C for 2–3 months to enhance hatching. For inoculum, J2s were released from the cysts by crushing and were adjusted to a concentration of 600 to 700 juveniles/ml.

Pre-germinated seeds of each accession was transferred to polyvinyl chloride (PVC) pipes (16 cm in height × 2.5 cm in diameter) filled with sieved double steam sterilized soil. Ten days post transplantation each accession was inoculated with 1 ml solution of second stage juveniles (J2s) by making three holes in the soil next to stem base. The holes were covered with soil after inoculation. Hoagland’s solution was used to fertilize the plants as needed throughout the experiment. Cysts were extracted from both root and soil 75 days post inoculation as reported in the literature^42^. Cysts were counted for all accessions under a stereo-microscope.

### Statistical analysis

The ANOVA was carried out using the *Agricolae* package in R program and descriptive statistics such as mean, median, standard deviation were calculated for cyst count using *Pastecs* package in R program^43^. For describing and presenting the phenotypic data, violin plots were prepared for two different environments as well as for combined environment (E1, E2 and CE) with the help of ‘*vioplot*’ package in R program ^43^.

### Population structure analysis

To infer the population structure, principal component analysis (PCA) was conducted through Genomic Association and Prediction Integrated Tool (GAPIT) package^44^. First two PCs were used to demonstrate the 2D plot which shows the distribution of accessions into different sub-groups.

To reveal the population structure of 180 wheat accessions the STRUCTURE v.2.3.4 software was used^45^. A set of 12,908 SNP markers were used to perform this analysis. Three independent runs for each *K* value from 1 to 10, where *K* is the number of subpopulations, were carried on the basis of an admixture model and correlated allele frequency. The length of burn-in period was set at 5,000 while the number of replication of burn-in period was set at 50,000. To obtain the appropriate *K* value, the normal logarithm estimated for the probability of fit, which in average of ten runs, provided in the output result of STRUCTURE was plotted against *K*. The value of *K* reaches the plateau once the minimal number of groups describing the best population structure has been achieved^45^. To predict the real number of subpopulations, an ad-hoc quantity statistic (Delta *K*) was used which is based on the rate of change in the log probability of data between successive *K* values^46^.

### Linkage disequilibrium analysis

Linkage disequilibrium analysis (LD) was carried out by using a set of mapped (N = 11,126) SNP markers with known positions. LD between markers was calculated as squared allele frequency correlation estimates (r^2^) using TASSEL v.5.0. Genome-wide and intra-chromosomal quantification and graphical representation of LD decay was generated using R program ^43^ by plotting the squared correlation coefficients (r^2^) vs. map distances in centiMorgans (cM). The 95^th^ percentile of r^2^ values of unlinked loci was assumed to be the population-specific critical value of r^2^, beyond which LD was likely considered to occur due to genetic linkage.

### Genome-wide association analysis

Association mapping was performed using three multi-locus models viz. Multi loci mixed linear model (MLMM), Bayesian-information and linkage-disequilibrium iteratively nested keyway (BLINK) and Fixed and random model Circulating Probability Unification (FarmCPU) and a single-locus model, Mixed linear model (MLM) available in GAPIT package. Circular Manhattan plot and combined quantile–quantile (QQ) plot were generated through *CMplot* package in R program. GWAS analysis was carried out for all the environments (E1, E2 and CE). For conducting GWAS, the default significant threshold value implemented in GAPIT was set at FDR < 0.05 but this value is quite stringent and conservative and we could not identify any significant MTAs for the given trait. Therefore, MTAs were considered significant when P < 0.001 or −log10 (*p*-values) ≥ 3.0. MTAs detected in all the environments (E1, E2 and CE) were considered as stable MTAs.

The previously identified genes/QTLs/MTAs known for *H. avenae* resistance were also compared with the MTAs identified in the present study. For that purpose, physical positions of all the identified genes/QTLs/MTAs were obtained using Ensembl plant for *T. aestivum* and a chromosome map was prepared with MapChart software which represents all the genes/QTLs/MTAs identified on individual chromosomes.

### Candidate gene analysis

Sequence of significant MTAs were blasted against *T. aestivum* genome sequence information hosted at EnsemblPlants (http://plants.ensembl.org/Triticum_aestivum) to identify the candidate genes. All genes present within maximum window size of 50 kb before and after the marker were retrieved and studied for their function through the available literature. Selected genes known to play a role in disease resistance mechanisms were shortlisted and considered as potential candidate genes. The retrieved potential candidate genes were further submitted to the ‘Wheat Expression Browser-expVIP’ (expression Visualization and Integration Platform) (http://www.wheat-expression.com) for *in-silico* gene expression analysis^47,48^.

## Results

### Phenotypic evaluation and categorization of the accessions

**Figure 1:**
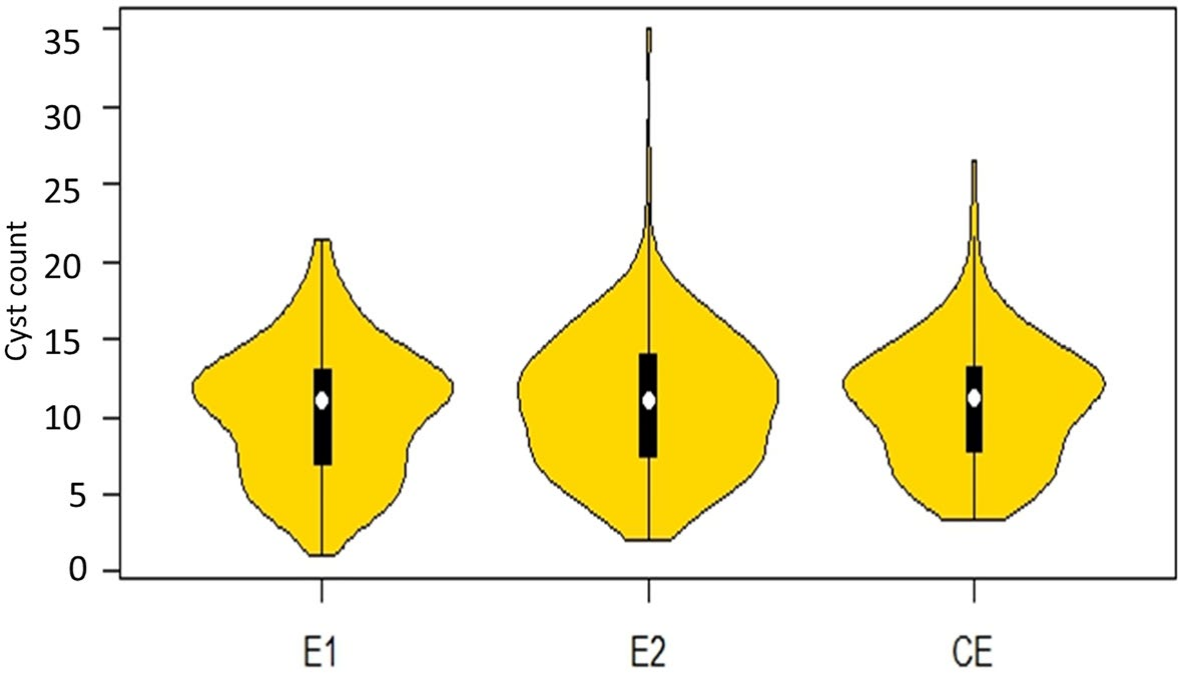
Violin plots of the distribution of cysts count in environment one (E1), environment two (E2) and the combined environment (CE). The vertical solid bar indicates the range of average values, and median is shown as a white circle, depicting the lower, medium and upper quartile.

Best linear unbiased predictor (BLUP) values were calculated for each of the two environments (E1 and E2) and across combined environment (CE) using Meta-R program V.6.0 in order to eliminate the environmental effect^49^. The frequency distribution of the phenotypic data of the two environments and combined (E1, E2 and CE) values are represented using violin plots **(**Fig. 1**)**. The distribution of cysts count in the violin plot indicated a high range of variation for nematode infection across the accessions. The mean value for cysts count ranged in between 3 to 27 cysts/plant.

Based on the BLUP values of the cysts from each plant, wheat accessions were categorized into five categories as resistant, moderately resistant, moderately susceptible, susceptible, and highly susceptible (Supplementary Table S2). Seven accessions namely TRI10703, NovaBanatka, NS74-95, Sonalika, ZG1011, TRI10704 and AlKanTzao had the lowest numbers of cysts count ranging from 3–4 categorized as resistant accessions. The three accessions namely TRI3570, TRI5332 and TRI4551 with cysts count ranging from 21 to 27 were considered highly susceptible. Another 53 accessions with cysts count ranging from 5 to 9 were categorized as moderately resistant. Set of 91 accessions with cysts count ranging from 10 to 14 were classified as moderately susceptible. The remaining 26 accessions were considered susceptible with cysts count from 15 to 19. The coefficient of variation (CV) and heritability (H^2^) was calculated as 37% and 93%, respectively (Supplementary Table S3). ANOVA results showed that the variations due to accessions and environments for the given trait were highly significant (Table 1).

**Table 1:** Analysis of variance (ANOVA), for number of cysts of *H. avenae* in 180 wheat accessions.

Source of variation	df	Sum sq	Mean sq	F value	Pr (> F)
Environment	1	117	116.63	26.705	4.03e−07***
Replication (within ENV)	3	231	77.01	17.634	1.14e−10***
Genotype	179	13,693	76.5	17.516	< 2e−16***
ENV × genotype	160	818	5.11	1.171	0.117
Residuals	344	1502	4.37		

Significant at ***0.001.

*df *degree of freedom, *Sum sq* sum of square.

**Figure 2:**
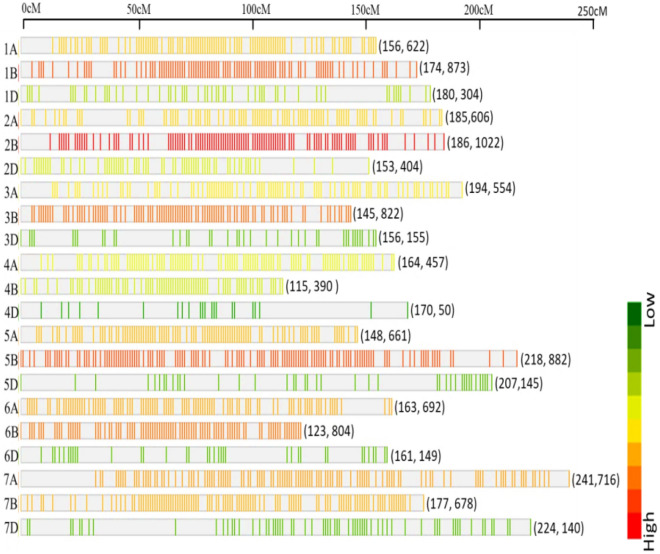
SNP density plot of the distribution of SNPs across all seven homoeologous chromosomes of wheat. The horizontal axis shows the length of the chromosome (cM); the SNP density is depicted by different colours.

Marker density and coverage on seven homoeologous chromosomes is shown in the Fig. 2. Maximum marker density was observed on B genome followed by A and D genomes. Also, within the chromosome, the highest number of markers was found on 2B while 4D chromosome have the lowest number of markers (Fig. 2). Sub-genome D has less coverage due to its lower contribution to hexaploid wheat diversity. Additionally, the peri-centromeric regions have low coverage, which is a common observation and is attributed to their low diversity^50–52^. Nevertheless, this may indicate that some regions of the genome remain uncovered, suggesting that a genome-sequence based approach such as exome-capture would be more appropriate.

### Population structure analysis

**Figure 3:**
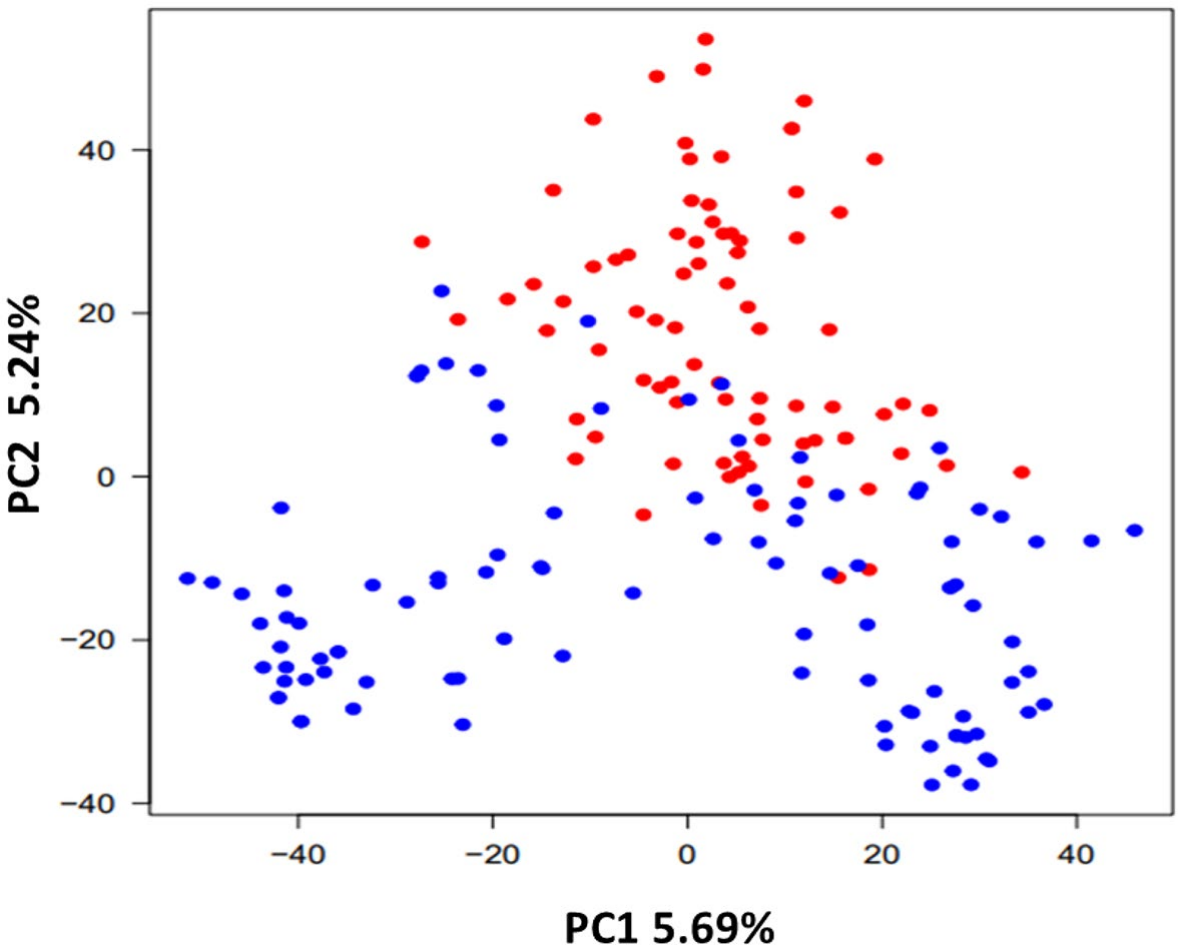
Principal component analysis (PCA) of 180 (100 spring and 80 winter) wheat accessions. The horizontal and vertical coordinates represent PC1 (with variance explained 5.69%) and PC2 (with variance explained 5.24%). Each dot represents a single accession. Spring and winter accessions are depicted by blue and red coloured dots respectively.

A PCA based test to check for the existence of possible clustering among accessions was performed (Fig. 3). Similar to a previous study^38^ majority of winter wheat accessions were clustered together with absence of further sub-clustering. Two separate clusters were found for spring wheat accessions. European spring wheat accessions were mostly clustered together. Similarly, population structure analysis revealed *K* = 3 (Supplementary Table S4) structuring as given in Supplementary Fig. S1a based on Delta *K*-value (Supplementary Fig. S1b).

### Linkage disequilibrium (LD) assessment

The intra-chromosomal and genome-wide linkage disequilibrium assessment performed using adjacent (pair-wise) SNP loci clearly revealed rapid LD decay with the increasing genetic map distances. Estimated linkage disequilibrium across each chromosome and whole genome are shown in the Supplementary Figs. S2a, b.

### Genome-wide association analysis

**Figure 4:**
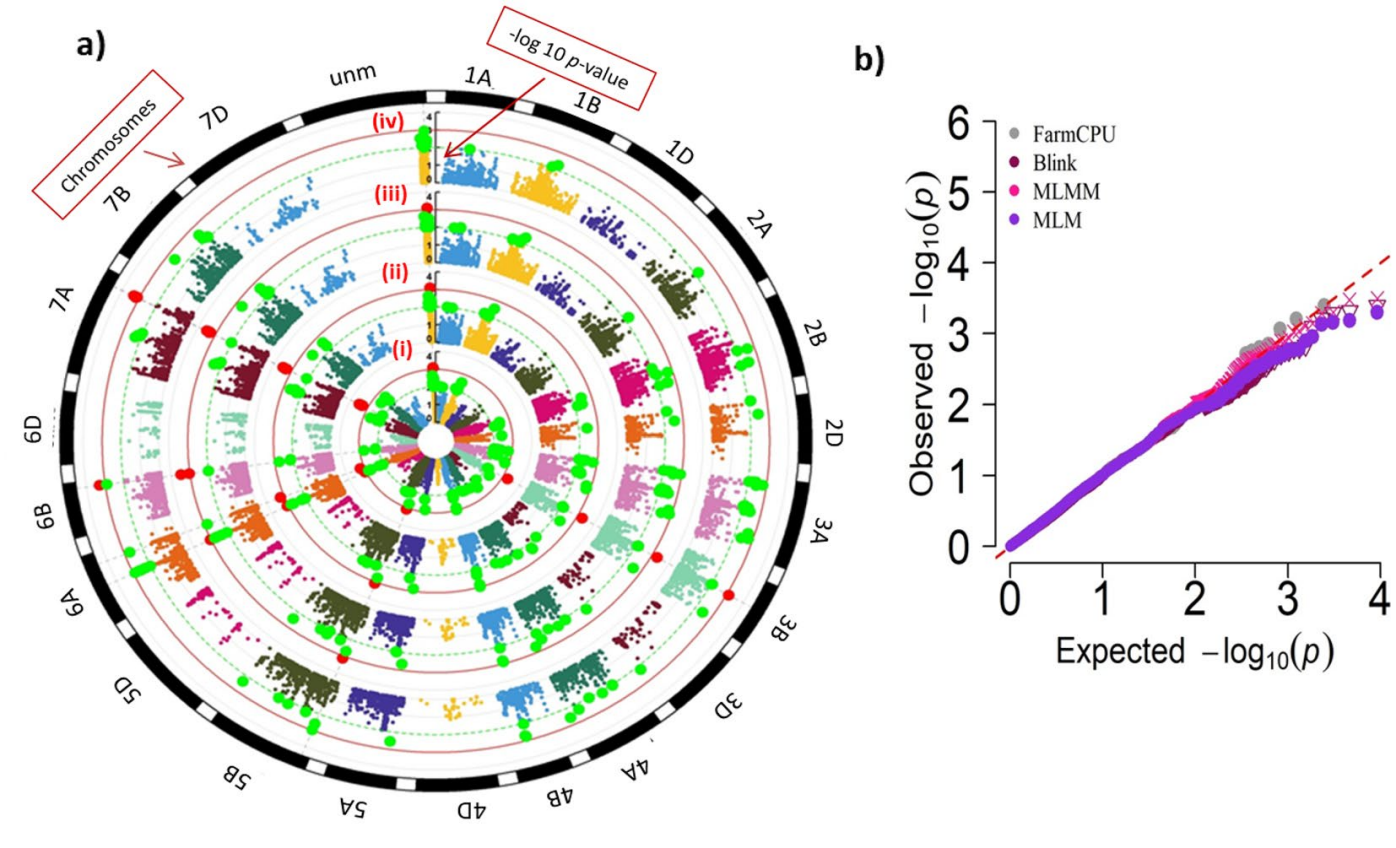
(**a**) Circular Manhattan plot representation of four different models used for GWAS analysis (i) BLINK, (ii) FarmCPU, (iii) MLM and (iv) MLMM (**b**) Combined QQ-plot of the observed and the expected *p*-values using different GWAS models.

**Figure 5:**
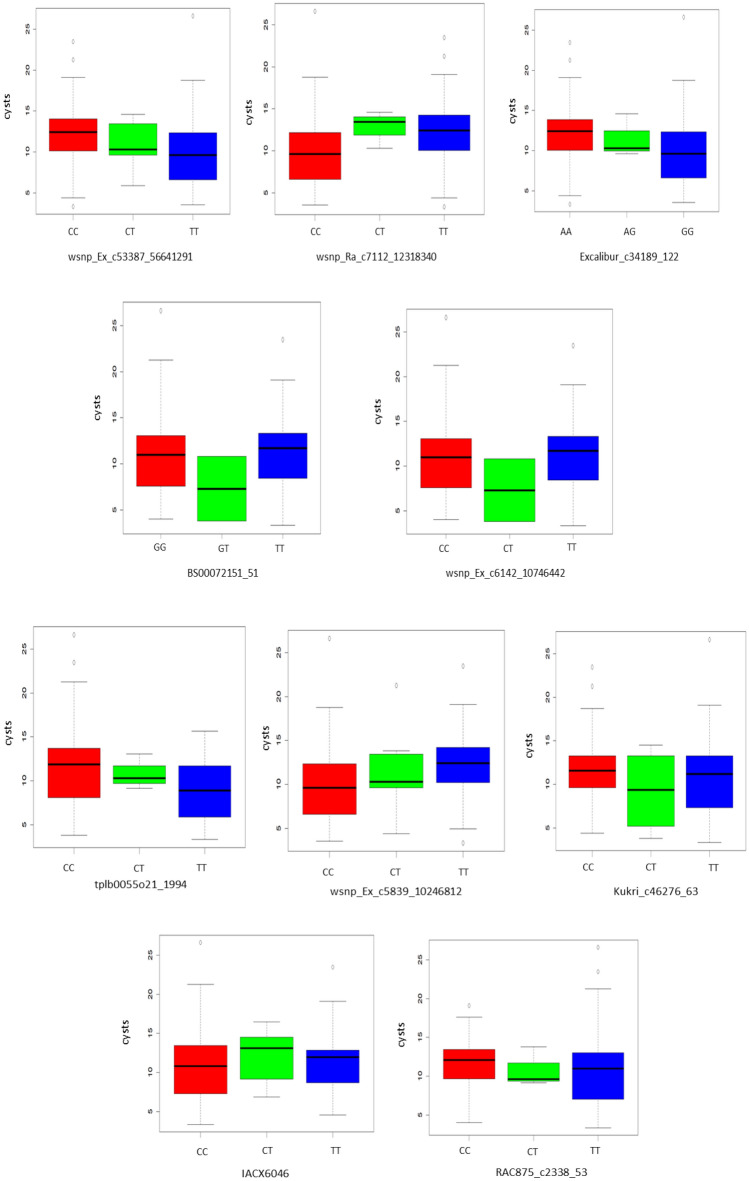
Boxplots showing the allelic effect of ten significant MTAs identified using all 180 wheat accessions and combined environment data, contributing to resistance or susceptibility for *H. avenae.* X-axis representing the marker name and alleles, Y-axis describes the cysts count.

Significant MTAs detected through different GWAS models were shown using circular Manhattan plot (Fig. 4a). Q–Q plots obtained for SNP results showed that the distribution of observed associations (*p*-values) were quite close to the distribution of the expected associations **(**Fig. 4b**)**. In total, 11 significant MTAs were detected through GWAS analysis of 180 wheat accessions (winter and spring growth habits) under combined environment (CE) of E1 and E2 **(**Table 2; Supplementary Table S5). These 11 MTAs were identified on chromosomes 7A, 6A, 6B, 3B, and 5B. The total phenotypic variance for the identified MTAs ranged from 2 to 16% (Table 2). The physical position of one of the highly significant unmapped MTA (*wsnp_Ex_c5839_10246812*) was obtained by performing simple BLAST of SNP sequence against the wheat genome sequence (IWGSC). This MTA was mapped on chromosome 7A. One of the significant MTA namely “*wsnp_Ex_c53387_56641291*” was detected in all environments and considered to be stable. Additionally, significant MTAs detected by more than one method suggest authenticity of these MTAs (Table 2; Supplementary Table S6). The allelic effect of the identified MTAs (10 out of 11) ranged from 1.06 to 1.40 (Table 2; Fig. 5) An allele leading to a decrease in cysts count was considered to be favorable alleles whereas unfavorable alleles were involved in increasing the cysts count.

**Table 2:** Details of eleven significant MTAs identified at threshold [−log10 (*p*-value) ≥ 3.0] using all 180 wheat accessions and combined environment (CE) data of E1 and E2.

MTA (environment)	Models	Chr	Pos (cM)	Location (bp)	*p*-values	MAF	FDR adjusted *p*-values	Effect	R^2^
wsnp_Ex_c53387_56641291 (E1, E2, CE)	1, 2, 3, 4	7A	213	7A:717,089,588–717,089,788	0.00	0.45	0.96	−1.21	0.16
wsnp_Ra_c7112_12318340 (E1, CE)	1, 2, 3, 4	7A	213	7A:717,040,885–717,041,085	0.00	0.47	0.96	1.21	0.16
Excalibur_c34189_122 (E1, CE)	1, 2, 3, 4	7A	211	7A:717,257,422–717,257,522	0.00	0.46	0.96	−1.17	0.15
BS00072151_51 (E1, CE)	1, 2, 3, 4	3B	67	3B:419,241,099–419,241,199	0.00	0.5	0.96	1.07	0.05
wsnp_Ex_c6142_10746442 (E1, CE)	1, 2, 3, 4	7A	213	7A:717,037,257–717,037,372	0.00	0.46	0.96	−1.16	0.15
tplb0055o21_1994 (CE)	3, 4	6A	136	6A:605,564,278–605,564,378	0.00	0.19	0.96	−1.40	0.04
wsnp_Ex_c5839_10246812 (CE)	2, 3, 4	unm	unm	7A:717,036,667–717,036,867	0.00	0.44	0.96	1.14	0.15
Kukri_c46276_63 (CE)	2, 3, 4	5B	20	5B:16,002,367–16,002,467	0.00	0.34	0.96	−1.06	0.04
IACX6046 (E1, CE)	2, 3, 4	6A	85	6A:538,056,638–538,056,757	0.00	0.37	0.96	1.24	0.05
RAC875_c2338_53 (E2, CE)	1, 2	6B	109	6B:704,183,485–704,183,585	0.00	0.26	0.96	−1.27	0.04
Tdurum_contig76116_469 (E2, CE)	2	6B	109	6B:704,038,802–704,038,899	0.00	0.24	0.9	NA	0.02

1, 2, 3, 4 represents MLM, MLMM, BLINK and FarmCPU respectively.

*Chr* chromosome, *Pos* position, *cM* centimorgan, *bp* base pair, *unm* unmapped, *NA* not available.

**Figure 6:**
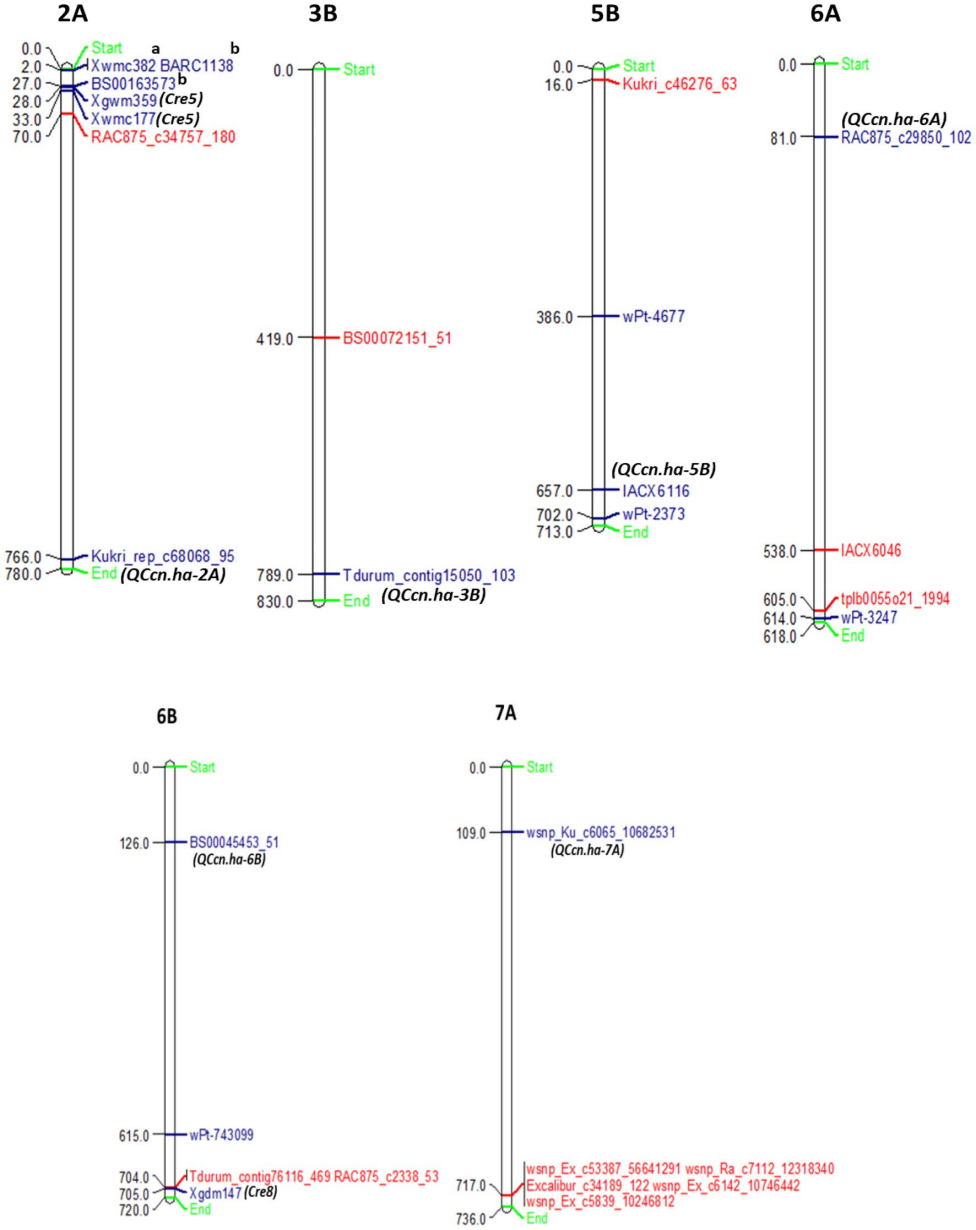
Chromosome maps showing MTAs detected in the present study and genes/QTLs/MTAs of *H. avenae* reported in the previous studies. The MTAs detected in the present study are depicted in red font. Markers associated with previously reported genes/QTLs/MTAs for *H. avenae* are shown in blue coloured font. The designated genes/QTLs associated with the markers are shown in black coloured font. Superscript a: represents the QTL, *QCre.pau-2A* linked to marker Xwmc382 and superscript b: represents the QTL, *QCre-ma2A* linked to marker BARC1138 and BS00163573 respectively.

Physical positions of all 11 MTAs were compared with the previously identified genes/QTLs/MTAs for *H. avenae* resistance resulting in the detection of MTAs either in vicinity or coinciding with genes/QTLs/MTAs already known for *H. avenae* resistance (Fig. 6**)**.

### Identification and in-silico expression analysis of putative candidate genes

**Figure 7:**
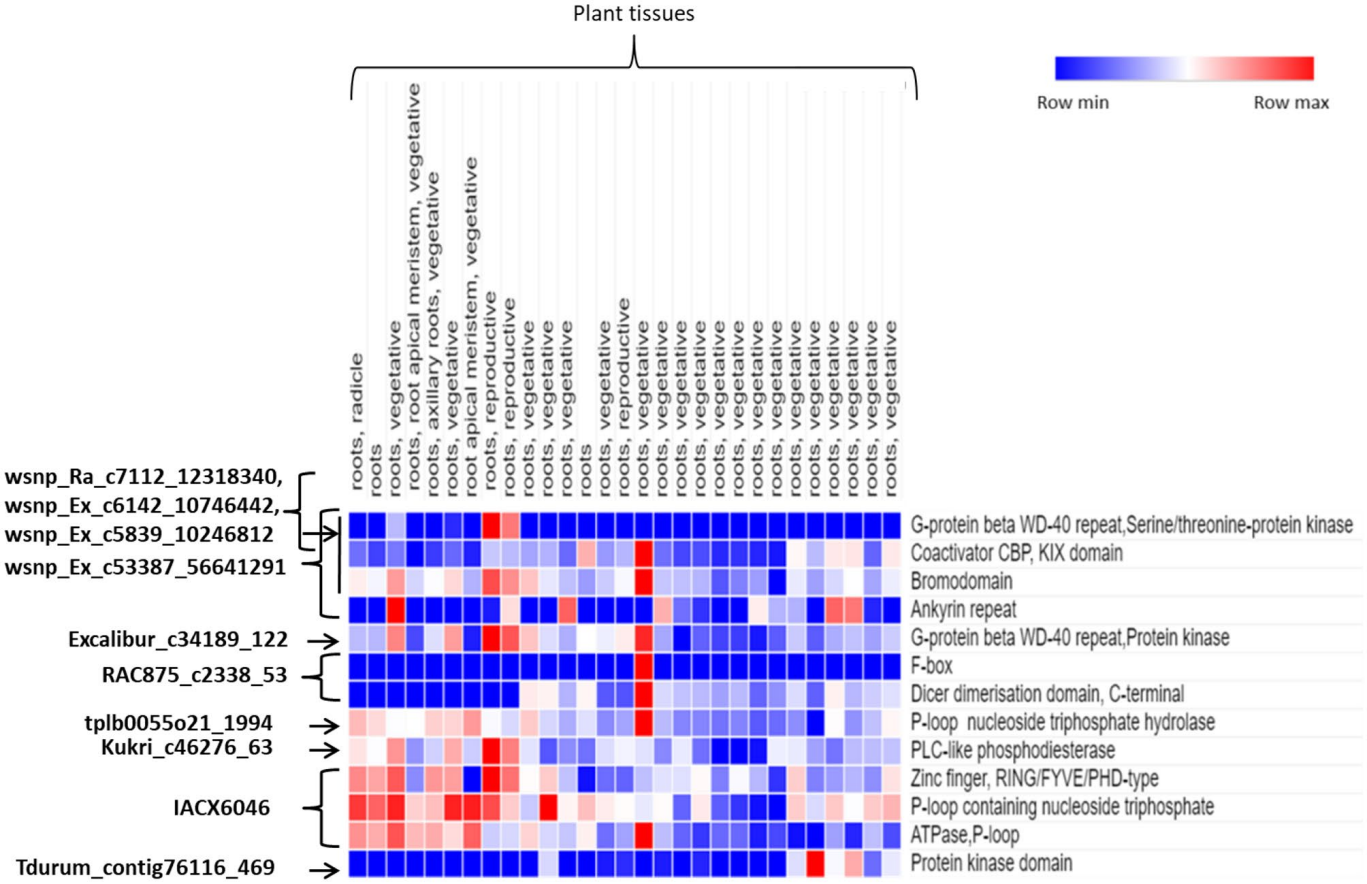
Expression profiles of candidate genes obtained in 180 wheat accessions under CE (combined environment) in wheat roots. Blue, white and red indicate low, medium, and high expression, respectively.

Out of 11, only ten MTAs located on different chromosomes were linked to putative candidate genes (Supplementary Table S7). No hit was found for MTA *BS00072151_51* located on the chromosome 3B. We identified a total of 33 putative candidate genes for ten significant MTAs associated with *H. avenae* resistance (Supplementary Table S7); only 17 of these candidate genes, including F-box like domain superfamily, ankyrin repeat-containing domain, wall-associated receptor kinase galacturonan-binding, coiled-coil domain, serine threonine kinases domain, WD40s, Zinc finger RING/FYVE/PHD-type, etc., have a putative role in disease resistance (Supplementary Table S8). In-silico gene expression analysis of these genes from WheatExp database showed that majority of these genes were found to be differentially expressed in wheat roots (Fig. 7; Supplementary Table S8).

## Discussion

Identification of natural source of resistance against different PPNs is probably one of the most environment friendly and economically feasible method to identify sources of resistance against *H. avenae* in wheat. Very limited QTL  mapping and GWAS studies have been performed in the past to identify the source of resistance against *H. avenae* in wheat^14,28,31,32,37,53,54^. However, bi-parental mapping population suffers from limited resolution and sourced few alleles. Association mapping on the other hand is more efficient as it has higher resolution mainly due to many historical meiotic recombination events. In recent years, numerous algorithms and software have been developed to improve statistical power, computational efficiency, and to reduce false associations in the GWAS approach^55^. The present study is focused on the identification of novel genetic sources of resistance to *H. avenae* in wheat through GWAS approach. High variation was found for cyst counts (3–27 cysts/plant) across the screened wheat accessions which are in accordance with the results obtained by Dababat et al.^37^.

In the present study, clustering was observed in European spring wheat accessions that could be due to their local adaptation in Europe. It is not surprising, however, given that spring wheat is a major crop in other parts of the world, such as South–East Asia, where spring wheat is planted in the winter. LD analysis identified long-range LD (Supplementary Fig. S2a) with some marker pairs having larger LD values than others which is in accordance with the previous studies on European wheat germplasm^50,51^. Besides this, we found no preference for finding resistance in spring or winter accessions. As a result, we took the approach of using multiple GWAS model rather than a single model as robust in picking the signals confidently.

We identified 11 MTAs and some of these MTAs are located nearby to or co-localized with genes/QTLs/MTAs already known for *H. avenae* resistance as shown in the Fig. 6. On the distal end of the chromosome 6BL, a major gene *Cre8* linked to marker Xgdm147 has been identified using a double-haploid (DH) population^11,31^. *Cre8* is well known in imparting resistance against CCNs by reducing nematode counts^11^. The presence of *Cre8* locus near the distal end of chromosome 6BL was further confirmed by using DArT, SSR, EST-based and SNP markers on same mapping population^32^. Seven molecular markers, closely linked with *Cre8,* were recommended for use in marker-assisted selection of the *Cre8* resistance locus in wheat breeding^32^. In the present study, we also identified two significant MTAs (*Tdurum_contig76116_469* and *RAC875_c2338_53*) on chromosome 6BL which are co-localized with the *Cre8* gene (Fig. 6). This result further supports the previous finding where *Cre8* associated genomic region decreased the cysts count significantly.

Short arm of chromosome 2A carries *Cre5* gene, from *Ae. ventricosa,* that is very effective against *H. avenae*. This gene is widely known to confer resistance to *H. avenae* pathotypes worldwide like in France^56^, Australia^18^, Spain^57^, and to uncharacterized pathotypes from the Pacific Northwest (PNW) of the United States^58^. *Cre5* is found to be linked with Xgwm359 (28 Mb) and Xwmc177 (33 Mb) markers^31^. Another QTL resistance to *H. avenae* was also detected on 2AS (*QCre.pau-2A*) in a 4.0 cM marker interval BE498358–Xwmc382 by Singh et al.^54^. Cui et al.^33^ also reported a QTL *Qcre-ma2A* conferring resistance to *H. avenae*, with flanking markers BARC1138 and BS00163573 on chromosome 2AS. It was found to be associated with the marker VRGA-F11/VRGA-R5, specifically for the 2NS translocation from *Ae. ventricosa* that harbours *Cre5* ^59^. The 2NS chromosome segment from *Ae. ventricosa* was transferred to the chromosome arm 2AS in VPM-1 wheat line^56^. Also, *Qcre-ma2A* is predicted to be *Cre5* because the 2NS chromosomal segment that harbours *Cre5* and other genes involved in disease resistance are from VPM-1^60^. In the present study, we also identified a MTA (*RAC875_c34757_180*) on chromosome 2AS located close to *Cre5* gene. However, significance level of this MTA is slightly below the threshold of -log10 (*p*-values) ≥ 3.0 that can be attributed to environmental effect. Two significant MTAs “*IACX6046”* and “*tplb0055o21_1994”* were observed close to the previously reported MTA *wPt-3247* on 6A chromosome^37^.

The sequences of the SNP loci were mapped on available bread wheat reference sequence and 17 candidate genes having role in disease resistance were identified. Protein domains related to plant resistance mechanisms detected in the present study are: (i) F-Box proteins, (ii) Ankyrin repeat-containing domain, (iii) Coiled-coil (CC) domain, (iv) RING/FYVE/PHD-type Zinc finger, (v) Serine threonine kinases and (vi) WD40s. (Supplementary Table S7). F-box like domain is known to play vital role in plant responses to environmental stresses and plant defense responses^61–64^. The F-box protein such as COI1 has been known to implicate in jasmonate-regulated defense responses^61–63,65^. It was earlier reported that several ankyrin proteins in plants are strictly involved in protein–protein interactions and may also play vital roles in plant immunity^66,67^. *YrU1* (a stripe rust resistance gene) from the diploid wheat (*Triticum urartu*) is an nucleotide-binding leucine-rich repeat receptor (NLR) and its ankyrin domain may get interacted with other NLR proteins or can act as an integrated decoy domain thereby interacting with the effector of the pathogen resulting in increasing plant immunity. Other ankyrin proteins such as ACD6 and BDA1 are positive regulators of salicylic acid (SA) signaling in defense responses^68–70^. Brown plant hopper-resistance gene *BPH14*, a CC-NB-LRR (CNL) gene has been found to confer resistance to brown plant hopper (BPH) and white-backed plant hopper (WBPH)^71^. Its structural and functional analysis indicates that the CC and NB (nucleotide binding) domains confer BPH resistance and activate SA signaling pathways. A putative RING/FYVE/PHD-type zinc finger gene is engaged in programmed cell death and also shows a resistant response against pests^72^. Receptor serine-threonine kinases (RSTK) generally interact with different other proteins and are involved in disease resistance^73^. TaWD40s were involved in responses to several stresses such as cold, heat, drought, and also powdery mildew infection^74^.

## Conclusion

Current study identified 11 significant MTAs conferring *H. avenae* resistance in wheat. The wheat accessions showing resistant against CCN infection and significant MTAs provide valuable resources to CCN resistance wheat breeding programme. The next step is to develop high-throughput marker assay such as Kompetitive Allele Specific-PCR (KASP) from novel MTAs as well as MTAs overlapping with previously known QTLs. Genomic regions with MTAs detected in the present study and previously known QTLs/MTAs shall be targeted for *H. avenae* resistance breeding in wheat. Moreover, resistant MTAs that co-localized with genes like *Cre5* and *Cre8* may be more effective in transferring these genes to susceptible backgrounds. In a follow-up study we are generating large mapping populations from the identified novel regions for genetic cloning these CCN resistances.

## Supplementary Information

Supplementary Figures.

Supplementary Tables.

## Data Availability

All the data generated or analysed during the current study were included in the manuscript and its additional files. The raw data is available from the corresponding author on reasonable request. The collection of plant materials used in current study complied with institutional, national, or international guidelines.
