# Supplementary material for: Identification of genomic regions associated with cereal cyst nematode (Heterodera avenae Woll.) resistance in spring and winter wheat
Source: Sci Rep. 2023 Apr 11;13:5916. doi: 10.1038/s41598-023-32737-8 (PMC10090075; doi:10.1038/s41598-023-32737-8)
Supplement: Supplementary file 1 — Supplementary Figures. [file 41598_2023_32737_MOESM1_ESM.docx]

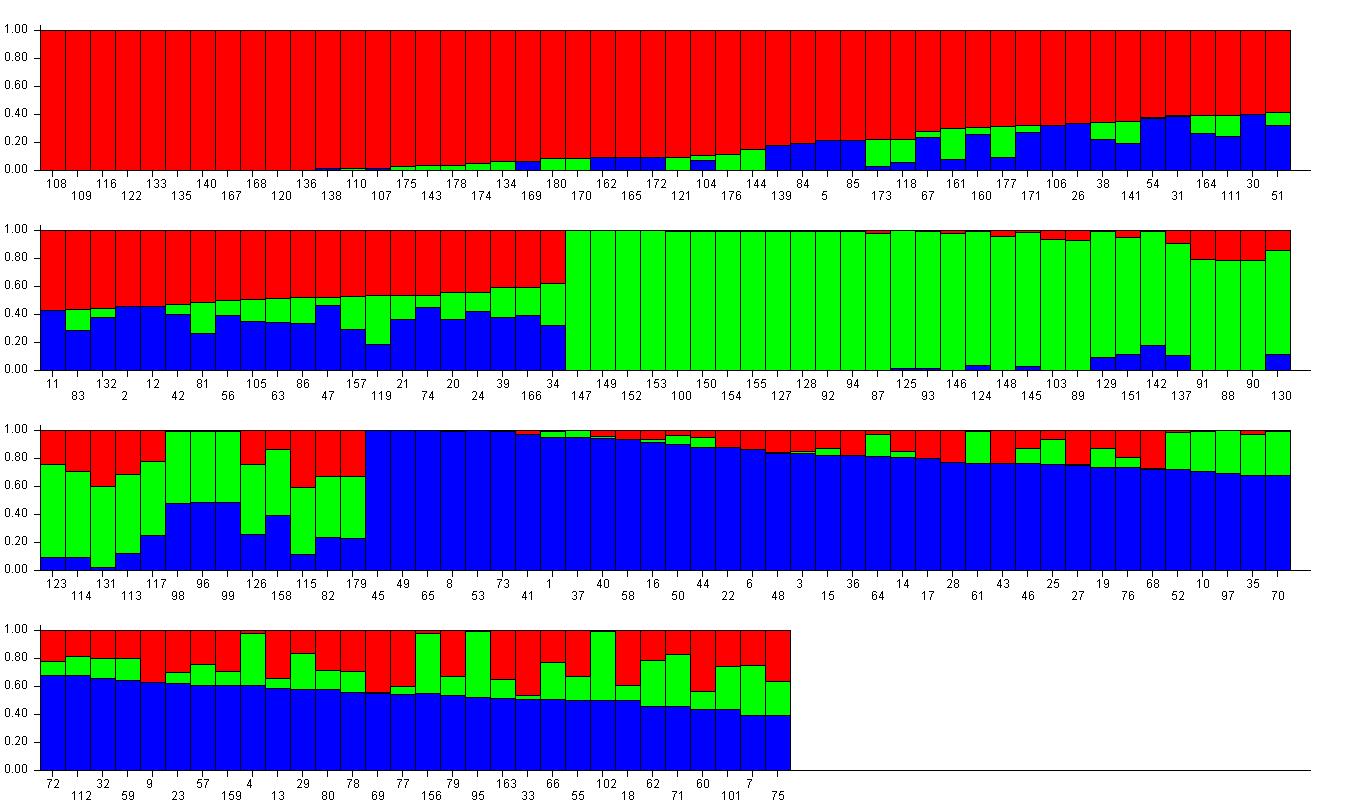


**Spring Wheats**

**Winter Wheats**


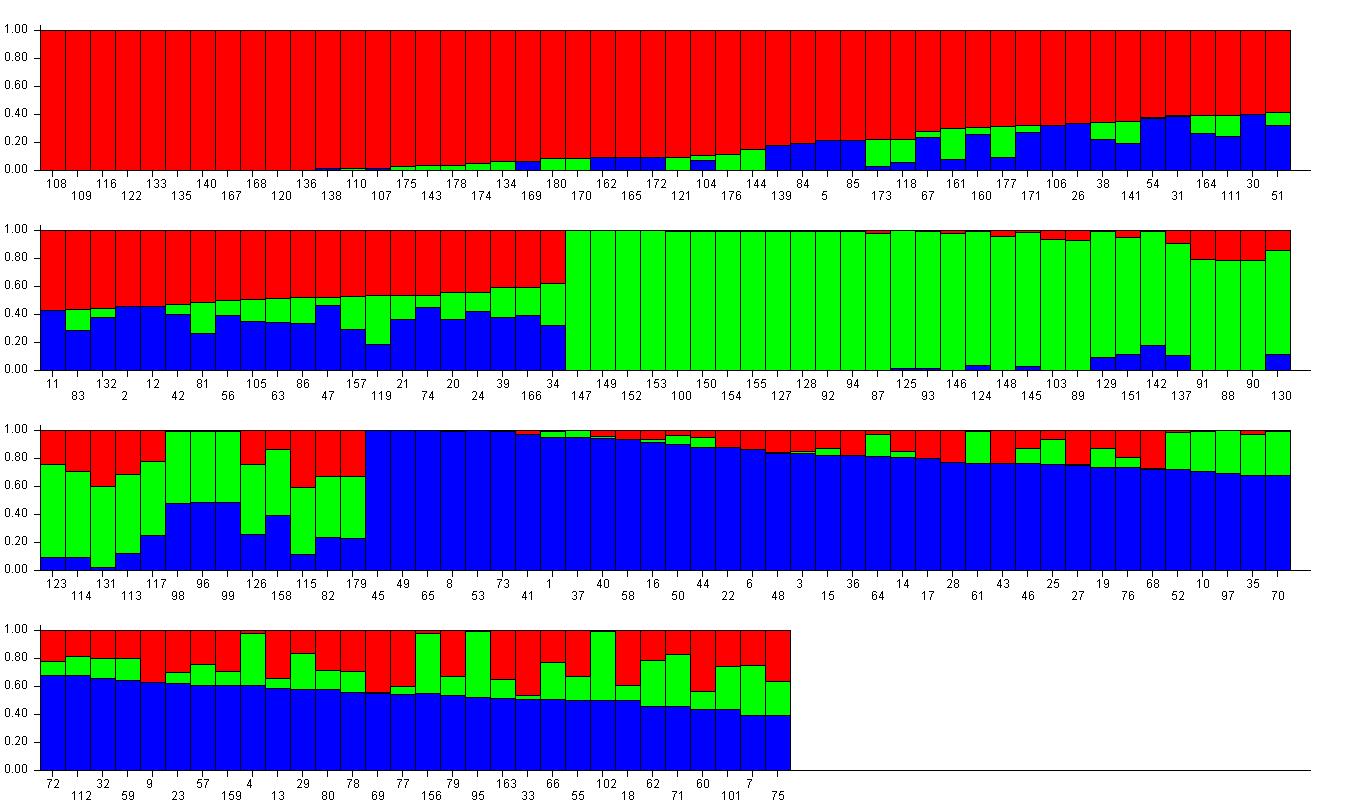


**Winter Wheat**

**Spring Wheat**

**Spring Wheat**

**Admixture**

**Admixture**

**Admixture**

**Admixture**

**Supplementary Figure S1a.** Estimated population structure of 180 wheat genotypes on (K = 3) using STRUCTURE. Population structure analysis revealed presence of three subpopulations within these 180 wheat accessions. Similar to PCA results, spring wheat panel was grouped into two different groups, as depicted by two different colours (red and green) with one group consisting of only European spring wheat accessions. Admixtures between two spring wheat types and spring with winter types were also present.

**
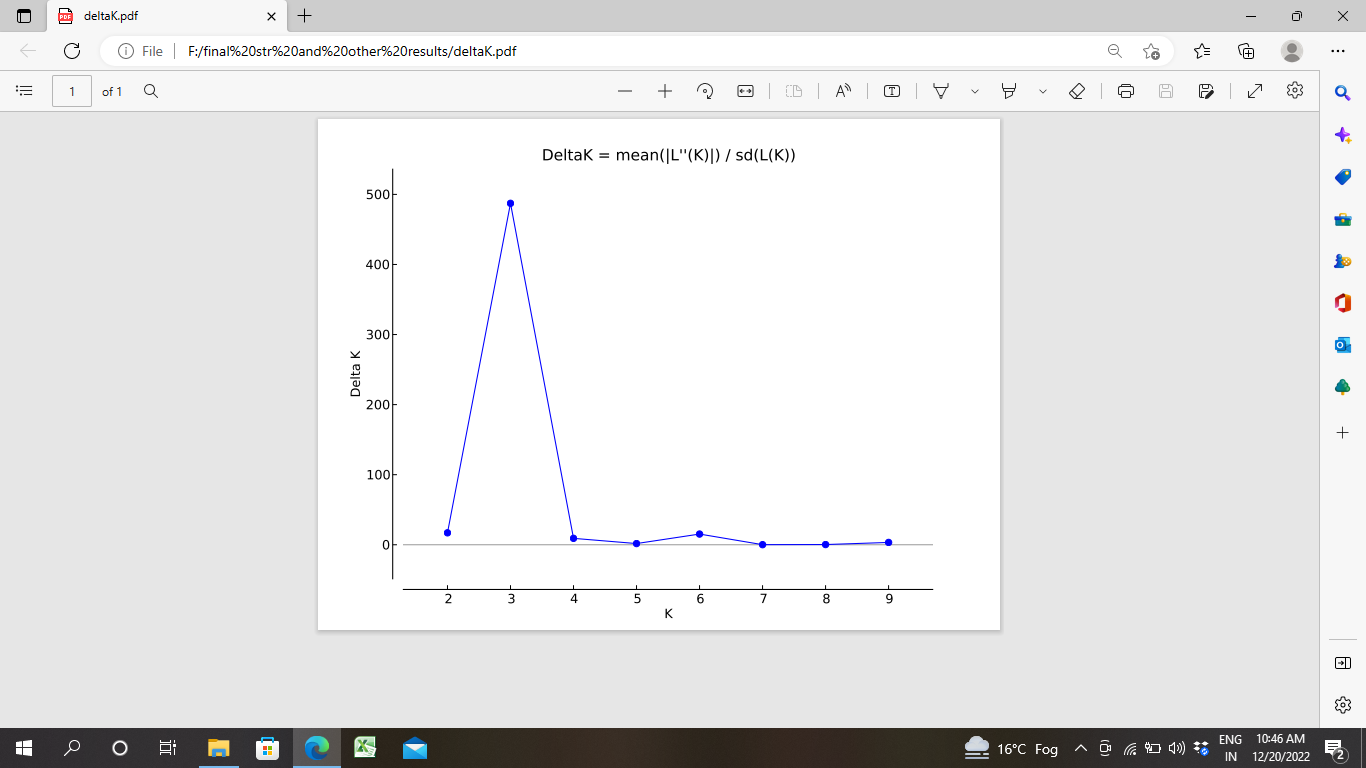
**

**Supplementary Figure S1b.** The ad-hoc quantity which is based on the second order rate of change in the log probability (Delta *K*) showed the peak at *K*=3.


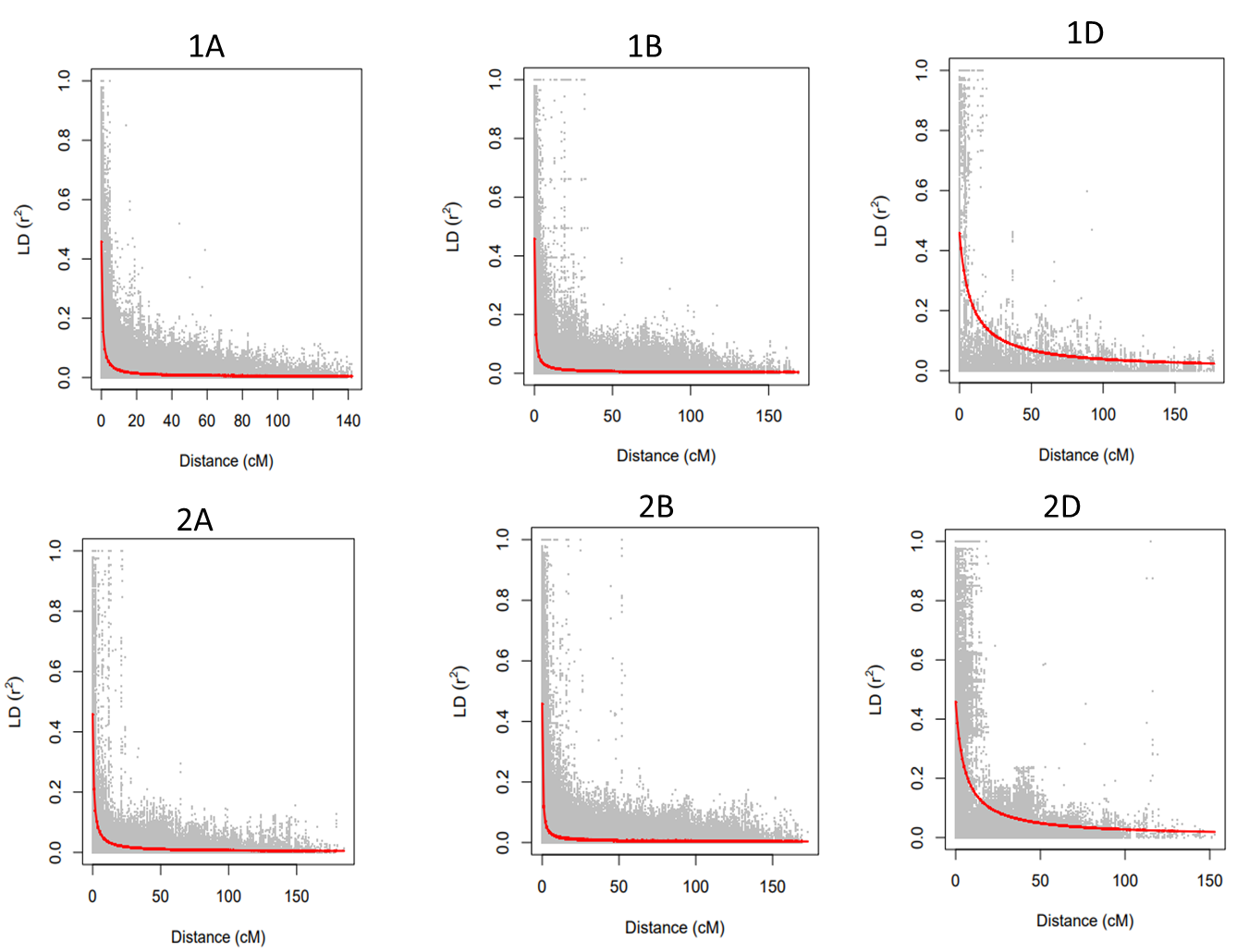


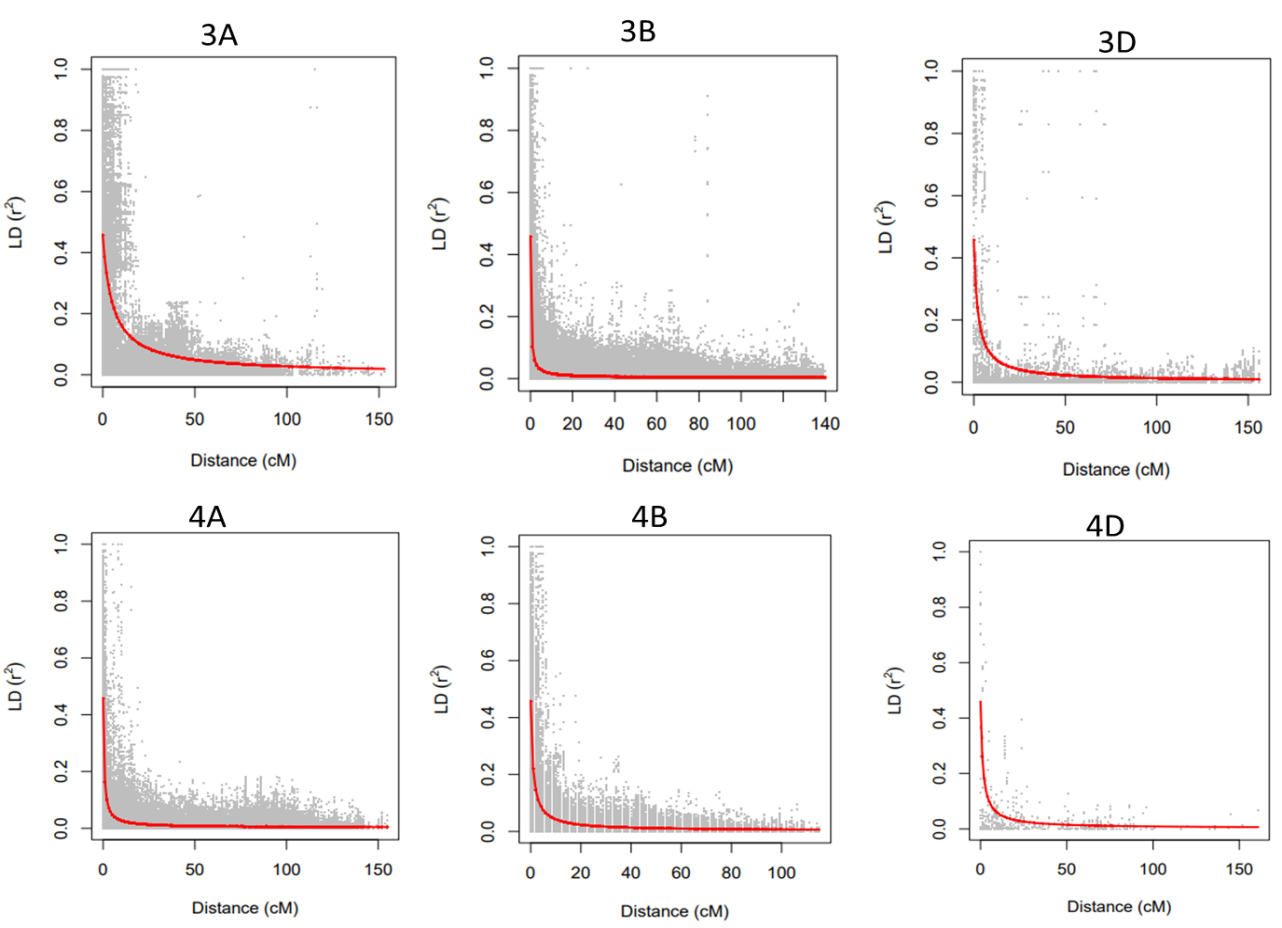


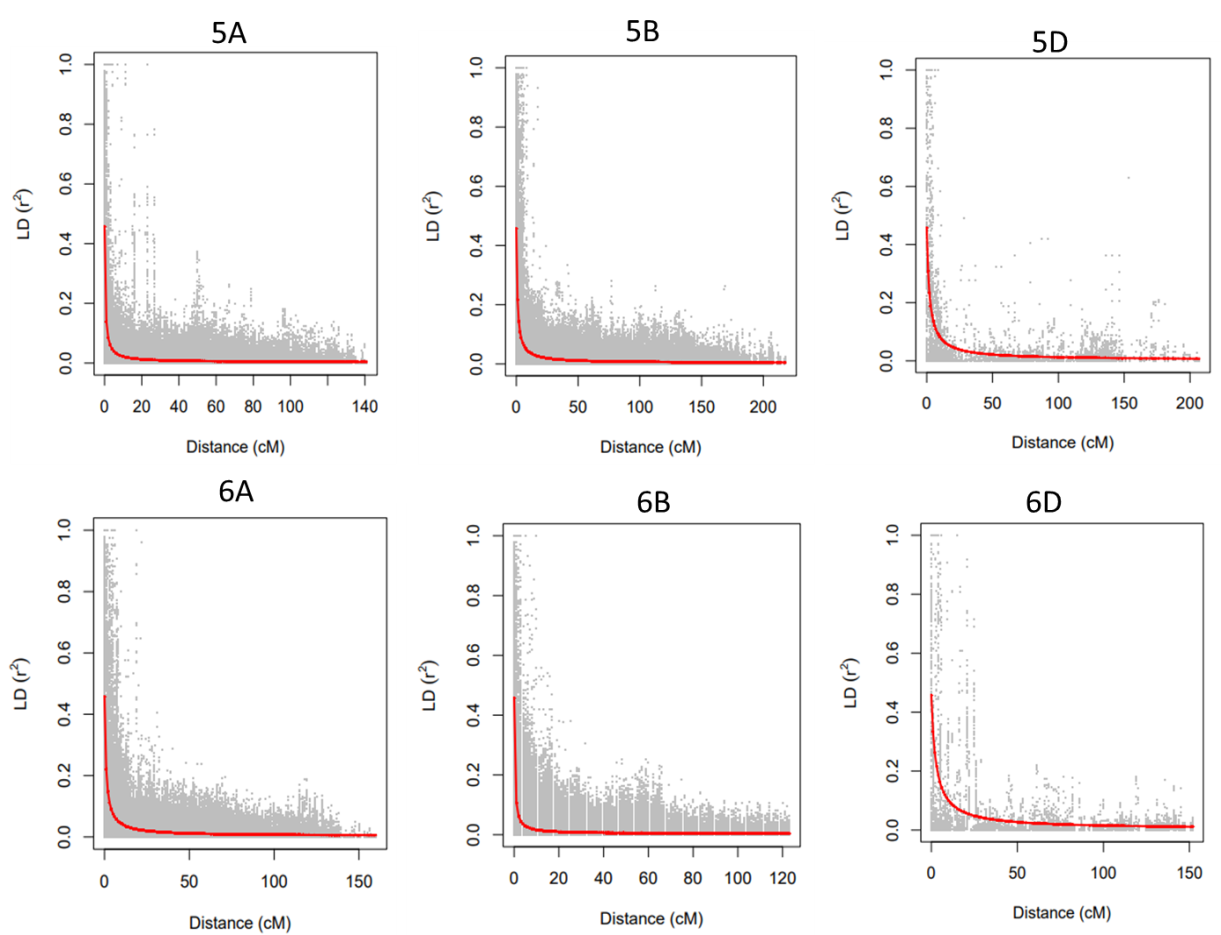


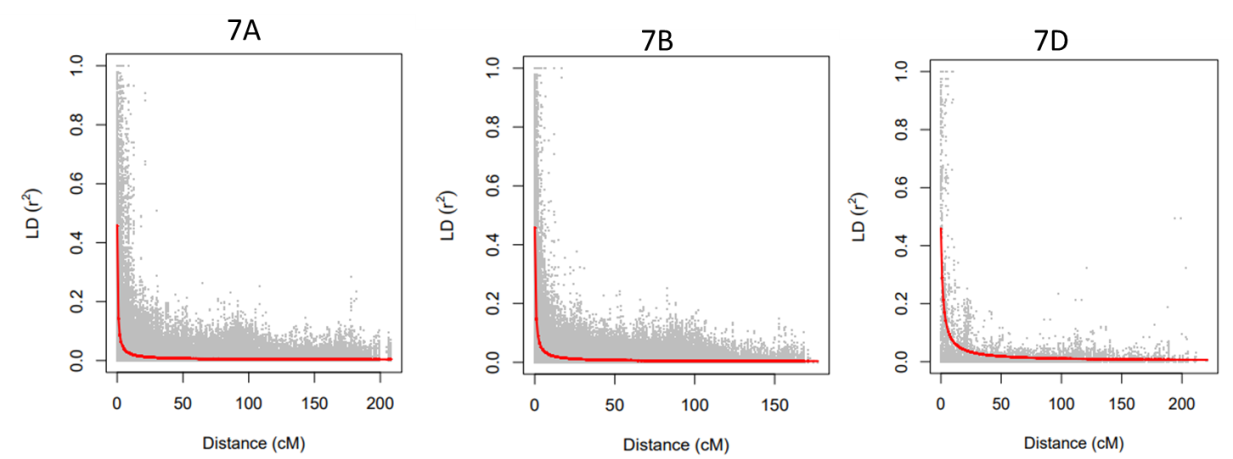


**Supplementary Figure S2a.** Linkage Disequilibrium (LD) plots in 180 (winter + spring) wheat panel. Intra-chromosomal plots of LD decay show r^2^ against the genetic distances (cM) between pairs of SNP loci.


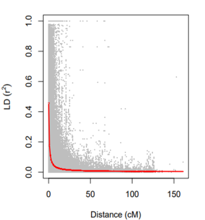


**Supplementary Figure S2b.** Genome-wide Linkage Disequilibrium (LD) plots in 180 wheat accessions.
